# Unraveling the complexity of skeletal dysplasias in the national health system

**DOI:** 10.3389/fendo.2025.1523737

**Published:** 2025-03-10

**Authors:** Dorra Najjar, Aleš Maver, Ana Peterlin, Helena Jaklič, Borut Peterlin

**Affiliations:** ^1^ Clinical Institute of Genomic Medicine, University Medical Centre Ljubljana, Ljubljana, Slovenia; ^2^ Laboratory of Biomedical Genomics and Oncogenetics, LR16IPT05, Pasteur Institute of Tunisia, Tunis El Manar University, El Manar I, Tunis, Tunisia

**Keywords:** CNV, diagnostic yield, molecular pathology, NGS, prenatal diagnosis, rare genetic diseases, skeletal dysplasia. skeletal dysplasia

## Abstract

**Introduction:**

Skeletal dysplasia (SD) is a large and heterogeneous group of rare genetic disorders that affects bone and cartilage growth. These disorders are diagnosed based on radiographic, clinical, and molecular criteria. However, the diagnostics is challenging due to clinical and genetic heterogeneity. We present the experience of systematic use of comprehensive genetic testing in the national health system and the molecular epidemiology of SD in Slovenia.

**Methods:**

We retrospectively reviewed 470 patients with clinical features of SD, including prenatal, childhood, and adult patients referred for diagnostic genetic evaluation to the national genetic reference center over ten years. In 262 patients, whole exome or whole genome sequencing was performed, while direct gene sequencing was performed in 208 patients with a specific clinical diagnosis.

**Results:**

A definitive genetic diagnosis using NGS was achieved in 50% (n=131) of patients. Among the positive cases, 49.61% initially presented with a nonspecific diagnosis of SD, and genetic testing contributed to establishing the diagnosis. Moreover, we demonstrated high genetic heterogeneity in our SD cohort with 66 distinct causative genes, resulting in different types of SD. In detail, we detected 132 causative variants, of which 29 were novel, which expanded the mutational spectrum of SD. Furthermore, pathogenic copy number variants (CNVs) were identified in 4.55% of the total number of variants, highlighting the importance of CNV analysis in expanding the yield of molecular diagnosis of SD.

**Conclusion:**

With the systematic use of WES and WGS, we have significantly improved the diagnostic yield of SD in the national health system and access to genetic testing. Moreover, we found significant genetic heterogeneity, and we report the genetic epidemiology of SD in the Slovenian population.

## Introduction

Bone tissue is a large compartment of the human body that implicates a tightly regulated process for developing, growing, and maintaining the human skeleton (cartilage and bone). SD is a clinically and genetically heterogeneous group of rare disorders that affects the process of bone and cartilage formation and homeostasis (1). Despite the individual rarity of these diseases, they present 5% of rare disorders (2), with a prevalence of at least 1/5000 births (3). SD has significant consequences on patients’ quality of life due to its high morbidity, and the most severe forms can reduce life expectancy (4, 5).

Recent advances in bone and cartilage metabolism have clarified the molecular causes of approximately 771 rare skeletal disorders, which exhibit highly complex phenotypes (6, 7). However, diagnosing this large group of disorders is challenging (8–10). Firstly, clinicians have limited knowledge of these rare disorders. Secondly, SDs exhibit significant genetic heterogeneity and pleiotropy. Thirdly, distinctive skeletal manifestations either do not appear until skeletal maturity or tend to diminish over time. Finally, some disease categories are still not well characterized. Moreover, these conditions often exhibit overlapping phenotypes, necessitating a multidisciplinary approach involving comprehensive evaluations by specialists across various fields. This approach combines diverse radiographic imaging techniques, clinical assessments, and, in some cases, histological evaluation from bone biopsies, resulting in a significant socio-economic burden on the healthcare system (11, 12).

A molecular diagnosis enables identification of the specific cause of the underlying SD; however, using hypothesis-based diagnostic approaches makes it challenging to determine which gene(s) should be investigated (7, 13). Additionally, the modes of inheritance are variable (including autosomal dominant, autosomal recessive, X-linked, and mitochondrial inheritance), and the mutational spectrum may encompass both CNVs and SNVs. This variability necessitates multiple tests, which are both time- and cost-intensive (7, 14). Presently, genetic testing is considered an essential element in the workup of patients with congenital SD because of the impact of these malformations on the quality of life of the patient and the risk of the lethality of some disorders. Genetic testing also facilitates primary prevention, family planning, and access to therapies or novel clinical trials for certain conditions with known genetic etiologies.

Recently, next-generation sequencing (NGS) has significantly advanced our understanding of the molecular pathology of SD, identifying over 552 genes involved in ossification, chondrogenesis, and bone metabolism (6). Nevertheless, translating novel genomic technologies into clinical practice has been slow. In this study, we present the genetic diagnostic strategy and evaluate the impact of systematically applying NGS to diagnose and gain insights into the molecular pathology and genetic epidemiology of SD in the Slovenian population.

## Materials and methods

### Patients

We conducted a retrospective study of 470 patients with a clinical diagnosis of SD, including prenatal, pediatric, and adult cases, who were referred for genetic diagnostics to the Clinical Institute of Medical Genetics at the University Medical Center Ljubljana between January 2013 and December 2022. All patients received genetic counseling and were referred by a clinical geneticist for genetic testing. We utilized NGS for genetic diagnosis in 262 patients. All patients underwent exome sequencing (ES) as the primary diagnostic approach, while for some families, trio design involving the parents, was performed for urgent cases, such as prenatal diagnoses, and Whole Genome Sequencing (WGS) was selectively performed for families with a strong suspicion of a genetic etiology, particularly in familial cases. For patients referred with a well-established diagnosis or a family history for specific diseases and variants, we used Sanger sequencing to identify common variants in specific genes, such as *FGFR3* (OMIM: *134934) for thanatophoric dysplasia type 1 (OMIM: #187600) or hypo/achondroplasia (OMIM: #100800 and OMIM: #146000), and *COL1A1* (OMIM: *120150) and *COL1A2* (OMIM: *120160) genes for osteogenesis imperfecta.

Patients’ data were obtained from the medical records, including the age of detection of the first clinical signs, clinical examination, standardized clinical diagnostic workup findings, associated phenotypic features, non-genetic laboratory testing, and family history. The clinical geneticist evaluated all these data, and the phenotypic features were described according to the Human Phenotype Ontology nomenclature (HPO) (15).

In this study, we analyzed retrospectively the results of genetic testing previously performed as a part of routine clinical diagnostics at our institution. Written informed consent was obtained from all patients. No genetic testing was performed solely for the purpose of this study.

### Next generation sequencing

ES was performed using a standardized series of procedures. Starting with an in-solution capture of exome sequences using various capture kits, including Nextera Coding Exome capture kit (manufactured by Illumina, San Diego, CA, USA) or Agilent SureSelect Human All Exon (V2, V5, V6) capture kits (manufactured by Agilent Technologies, Santa Clara, CA), Twist Human-Core-Exome kit (manufactured by Twist Bioscience Corporation, South San Francisco, CA, USA). As for the WGS, a standardized sequence of procedures was performed following PCR-free WGS library preparation protocol Illumina TrueSeq DNA Nano (manufactured by Illumina, San Diego, CA, USA). This was followed by sequencing on Illumina MiSeq, Illumina NextSeq 550, Illumina HiSeq 2500 Illumina, or Illumina NovaSeq 6000 platforms.

The data obtained from ES or WGS was subsequently analyzed using an internally developed pipeline based on the combined disease and phenotype gene target definition approach, as we previously described (16–18). Basic analysis for the detection and annotation of Single Nucleotide Variant (SNV), canonical splice site variants, and small indels (insertion/deletion) was performed according to the GATK Best Practices workflow (19–21). Moreover, we employed additional data analysis methods to expand the spectrum of detected genetic variation and improve the identification of causative variants in cases of negative results. To achieve this, we utilized the following approaches: (a) detection of noncanonical splice site variants (22, 23). (b) Annotation and analysis of mitochondrial sequence (c) CNV analyses (d) Identification of breakpoints was analyzed by detecting clusters of soft-clipped reads in aligned sequence, and (e) finally, the identification of repeat expansions throughout the genome and the long runs of homozygosity was analyzed (24, 25).

Only the variants classified as pathogenic or likely pathogenic were considered in estimating positive yield in this study. All the variants reported were inspected visually in the IGV browser. Low-quality variants and variants with GATK quality score below 500 were confirmed with Sanger sequencing (26). For CNVs, the confirmation was performed using different approaches: CGH array, multiplex ligation-dependent probe amplification (MLPA), and targeted PCR amplification.

### Gene selection

Based on human phenotype ontology (HPO) (17), OMIM (27), Pubmed databases (28), PanelApp (29) and ClinGen (30) tools, we created a targeted gene panel composed of 615 genes to identify relevant genes for our genetic investigation of SD. This method ensured the selection of relevant genes for our investigation, thus enhancing the accuracy of our genetic analysis by focusing on a small number of variants. For cases where no pathogenic variants were detected in the initial gene panels, we expanded our analysis to include all variants identified in the ES or WGS datasets.

### Classification

The pathogenicity of the variants was evaluated according to the standards and guidelines provided by ACMG/AMP (American College of Medical Genetics and Genomics and the Association for Molecular Pathology) and the Association for Clinical Genomic Science (ACGS) criteria (31, 32).

For the positive cases, the disease categories were classified according to the recent Orphanet classification of bone diseases (33).

## Results

### Genetic distribution and classification of the variants detected in our study

We retrospectively reviewed 470 patients with clinical features of SD, including fetal, pediatric (birth to 18 years), and adult (>18 years) patients who were referred for genetic investigation. We used NGS in 264 patients: ES for 244 patients, while trio-ES (11 cases) or WGS (7 cases) were performed on selected patients with negative results. The largest patient group consisted of pediatric patients (n=131), followed by adults (n=94), and 37 prenatal cases (37). Using NGS, we established a positive genetic diagnosis for 50% (n=131) of the referred patients. Of these, 38.93% were initially referred with an unspecified SD diagnosis, and 10.69% had been misdiagnosed. We identified 132 distinct causative variants across 66 different genes.

Conversely, we used Sanger sequencing for patients referred with a well-established diagnosis linked to a specific gene. We identified causative variants in 37 out of 208 patients (17.79%), detecting 26 pathogenic or likely pathogenic variants across 11 different genes (**Supplementary Table S1**).

**Figure 1:**
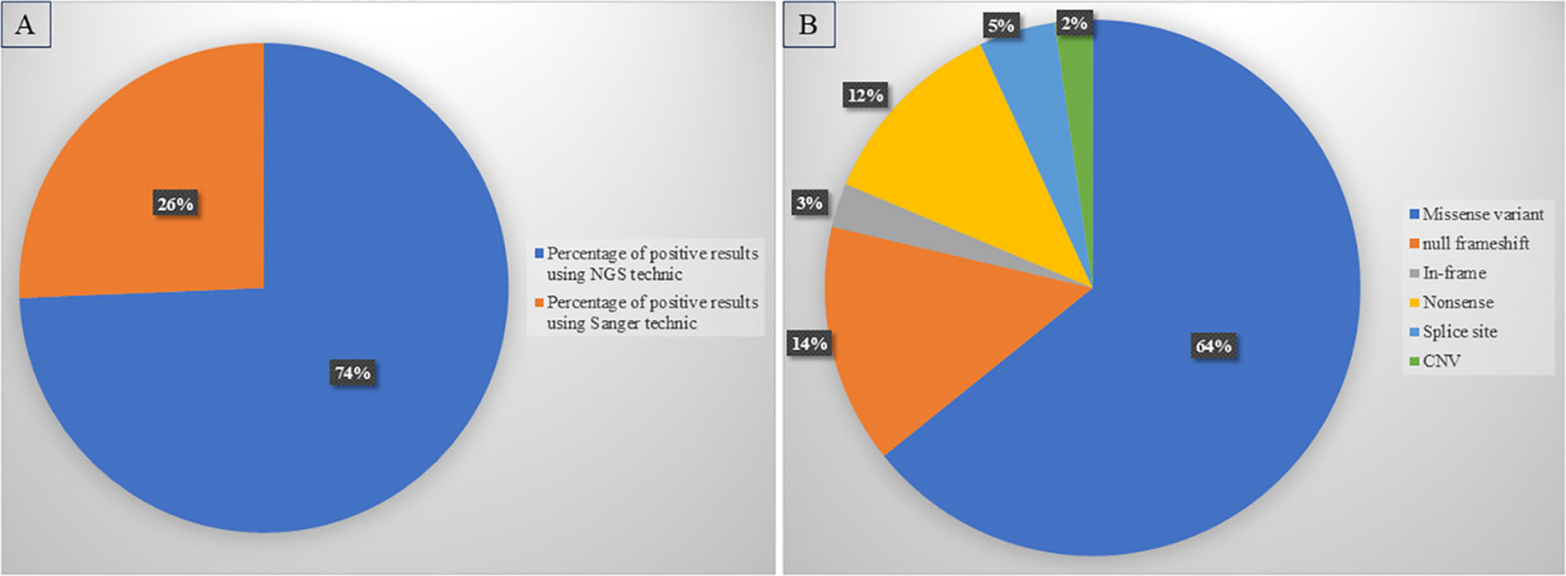
Diagnostic yield of diagnostic approaches and variant distribution. **(A)** High rate of positive diagnosis using NGS compared to Sanger sequencing. **(B)** Distribution of different types of variants detected in our cohort.

Comparing diagnostic approaches, we found that NGS identified 74% of the positive cases. (**Figure 1A**).

Missense variants were the most common in our cohort (64.22%), followed by null variants (30.86%); frameshift variants were the largest subgroup (14.45%), while nonsense variants accounted for 11.8% of positive cases, and splice site variants for 4.6%. Furthermore, in-frame variants (in-frame deletions or insertions) were detected in 2.65% of patients. In addition, CNV analysis identified 6 cases, increasing the total diagnostic yield by 2.27%. CNVs ranged from single-exon deletions or duplications to deletions or duplications of entire genes (**Figure 1B**).

Among all these variants, we identified 29 novel pathogenic variants associated with SD (**Supplementary Table S2**). Moreover, we identified ten variants classified as VUS in nine genes (**Supplementary Table S3**).

### Molecular pathology of SD diseases identified in our study

**Figure 2:**
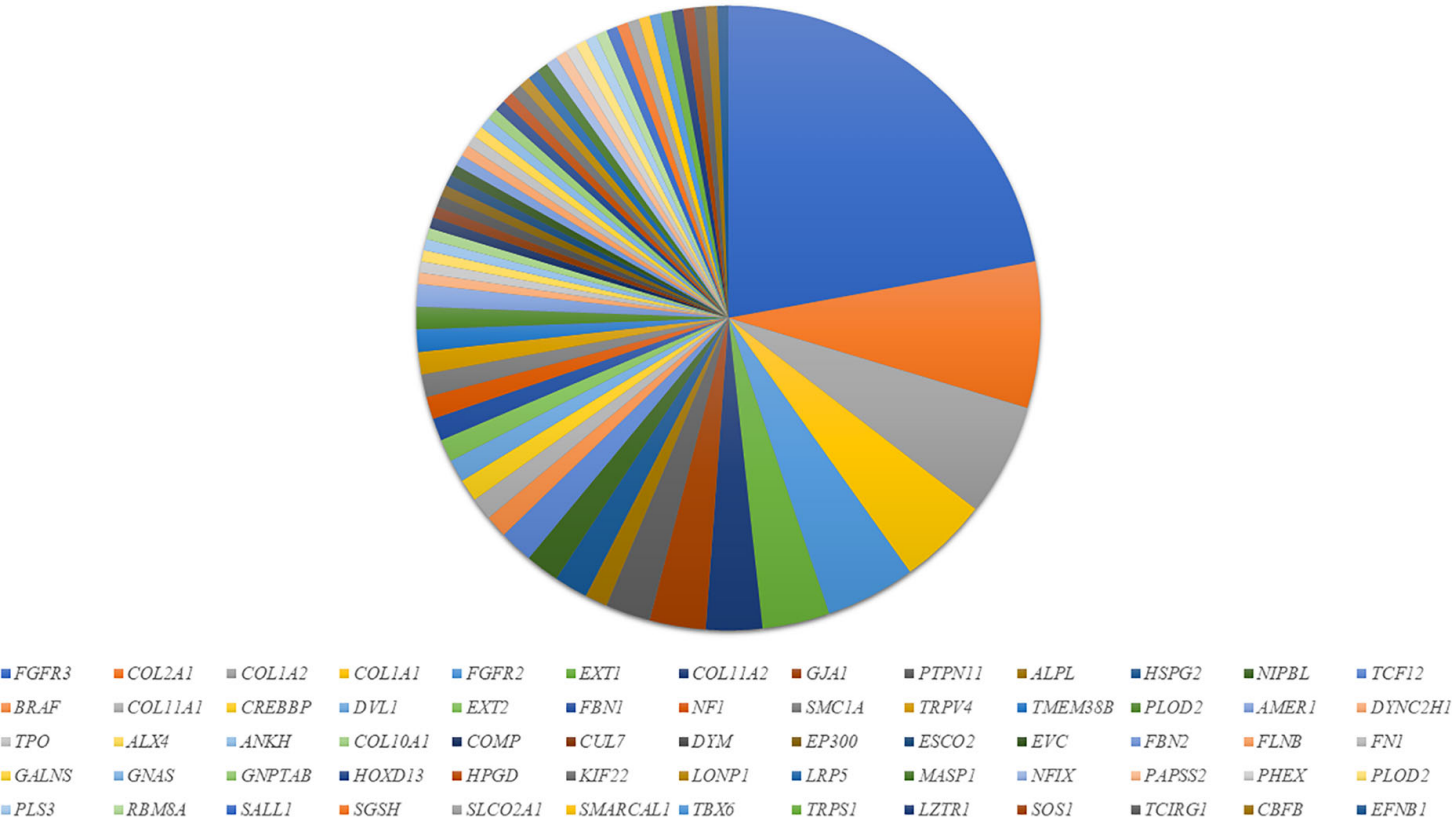
Genetic heterogeneity and distribution of the mutated genes in SD patients in our cohort.

We demonstrated considerable molecular heterogeneity in the Slovenian population, identifying 66 altered genes associated with various SD disorders (**Figure 2**). *De novo* variants in *FGFR3* (OMIM: *134934) were the most commonly identified, found in 30 patients with achondroplasia (OMIM: #100800) and hypochondroplasia (OMIM: #146000). Additionally, we detected *FGFR3* variants in five patients with Thanatophoric dysplasia (types 1 (OMIM: #187600) and type 2 (OMIM: #187601) and three with Muenke syndrome (OMIM: #602849). In addition, 20 patients were diagnosed with Osteogenesis Imperfecta (OI) (OMIM: #166210, #259420, #166220), with ten patients showing variants in the *COL1A2* gene (OMIM: *120160) and eight patients with variants in the *COL1A1* gene (OMIM: *120150). In the *FGFR2* gene (OMIM: *176943), five patients were diagnosed with Apert syndrome (OMIM: 101200), two with Crouzon syndrome (OMIM: #123500), and one with Pfeiffer syndrome (OMIM: 101600). Among patients with variants in the *COL2A1* gene (OMIM: *120140) (n=13), ten were diagnosed with spondyloepiphyseal dysplasia congenita (OMIM: #183900) and spondyloepimetaphyseal dysplasia (OMIM: #184250), two with Stickler syndrome (OMIM: #108300), and one with achondrogenesis type II (OMIM: #200610). Six patients had pathogenic variants in the *EXT1* gene (OMIM: *608177) causing hereditary multiple osteochondromas (OMIM: #608177).

### Novel genes and genotype/phenotype associations

We identified a null frameshift duplication c.295_296dup, p.(Pro100Leu fs*3) in the *CBFB* gene (Core-Binding Factor, Beta Subunit) (OMIM: *121360) associated with a new skeletal disorder resembling cleidocranial dysplasia syndrome (34).

The second variant c.2768T>G, p.(Leu923*), was detected in the *MIA3* gene (Melanoma Inhibitory Activity Family, Member 3) (OMIM: *613455), also known as *TANGO* gene (Transport And Golgi Organization Gene 1) in a fetus who presented with short bones of extremities (7 percentile), fibular aplasia, bilateral radial aplasia, tibial aplasia, hypoplastic nasal bone, delayed ossification, and congenital contractures. Variants in this gene were previously reported in a single case of Odontochondrodysplasia 2 with hearing loss and diabetes disease (OMIM # 619269).

### Diagnosis and classification of skeletal dysplasias detected in our cohort

**Figure 3:**
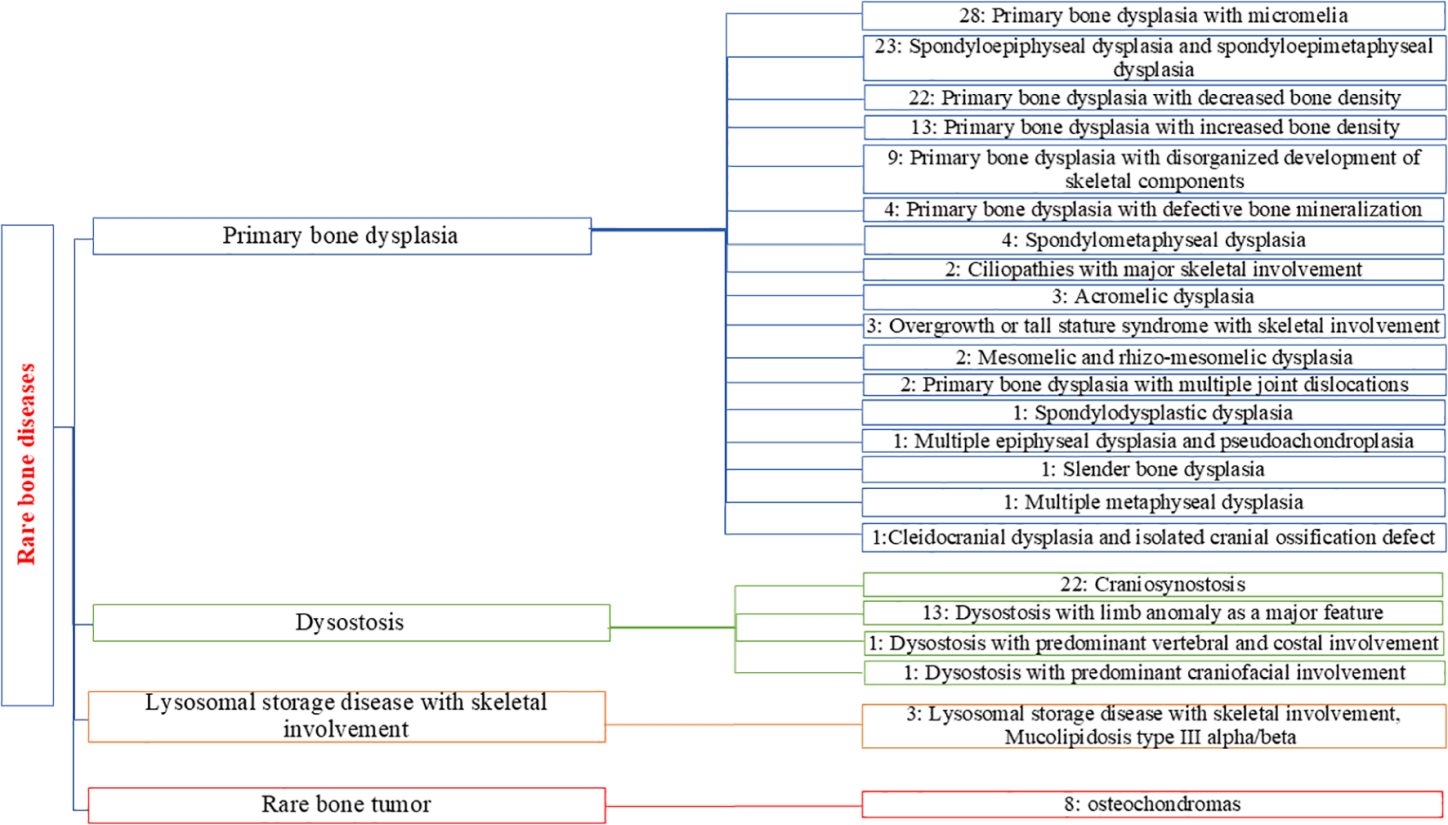
Distribution and classification of the subgroups of SD detected for positive cases in our cohort. The number associated with each subgroup represents the number of patients identified with positive genetic results.

We found significant clinical and genetic heterogeneity in our cohort, identifying different SD syndromes. To provide a clear overview of the prevalence and distribution of these various diseases, we used the Orphanet classification for rare bone diseases (ORPHA:93419). Each identified disorder was assigned to a specific group and subgroup, resulting in a comprehensive categorization of our findings into four groups. Primary bone dysplasias were the most common group of disorders among our patients (71.6%), followed by dysostoses (21.89%), rare bone tumors represented by hereditary multiple osteochondromas (4.73%), and lysosomal storage diseases (1.78%) (**Figure 3**).

The primary bone dysplasias represented the most diverse group, with 17 different subgroups identified. Primary bone dysplasia with micromelia was the most commonly diagnosed subgroup (n=28), followed closely by spondyloepiphyseal and spondyloepimetaphyseal dysplasias, which also formed a significant subgroup (n=23). We identified 22 patients diagnosed with primary bone dysplasia with decreased bone density. In the dysostosis group, craniosynostosis was the largest subgroup with the highest number of diagnosed cases (n=22), followed by dysostosis with limb anomalies as the second largest subgroup, with 13 diagnosed patients.

Among the diagnosed SD cases, autosomal dominant (AD) inheritance was the most common mode (82.99%), followed by autosomal recessive (AR) inheritance (14.72%) and X-linked dominant (XLD) inheritance (2.29%).

## Discussion

In this study, we present a systematic strategy for SD diagnostics in the Slovenian health care system (population of 2 million), which provides insight into molecular pathology and genetic epidemiology of SD.

SDs are clinically and genetically heterogeneous. The recent update of the Nosology of genetic skeletal disorders identified 552 genes associated with SD, which covers 95.6% of these disorders (6). Using NGS, we achieved a genetic diagnostic yield of 50% (131/262). This diagnostic yield is comparable to the findings of other studies in the literature, with an average diagnostic yield of 44.76% (10, 35, 36). The genetic spectrum of SDs is continuously expanding, as the genetic etiology of many disorders remains partially understood. New candidate genes and novel phenotypes are being discovered regularly (13). According to a recent recommendation by the American College of Medical Genetics and Genomics, ES or WGS should be utilized as a first- or second-tier test for patients with congenital abnormalities (37). Moreover, with the advancement of NGS, 67% of previously undiagnosed disease cases were resolved using ES (34), making it the most efficient diagnostic tool in terms of time and cost (35).

While our diagnostic approach primarily utilized next-generation sequencing, other genomic alterations may contribute to genetic disorders. Integrating additional methodologies, such as optical genome mapping, RNA sequencing, and methylation analysis, could enhance the diagnostic accuracy for rare diseases (38).

In this study, we identified 149 different pathogenic or likely pathogenic variants in 66 different genes associated with SD, underscoring the genetic heterogeneity present within our patient population. Using NGS, we identified 132 different variants across 66 genes, including 29 novel variants, which contribute to expanding genotype–phenotype correlations. Given that CNVs and mitochondrial variants have been reported to contribute to SD (5, 13), we systematically analyzed our SD cohort for these genetic variations. Using NGS, we achieved a diagnostic yield of 4.55% (n=6) for CNVs but did not detect any mitochondrial variants. Similar results were reported by scocchia et al. with 5.7% of positive results presenting CNV variants (35).

Throughout the course of our research, using ES, we identified two potential genes, *CBFB* and *MIA3*, associated with novel phenotypes of SDs. We recently published the *CBFB* gene alteration associated with a new skeletal disorder resembling cleidocranial dysplasia (34). Moreover, we detected a novel biallelic loss of function variant c.2768T>G, p.(Leu923*) in the *MIA3* gene in a fetus with a severe skeletal dysplasia phenotype. Homozygous variants in the *MIA3* gene were associated with Odontochondrodysplasia 2 with hearing loss and diabetes disease. This was discovered in one family presenting a synonymous variant in the *MIA3* gene leading to exon skipping associated with a skeletal dysplasia phenotype characterized by short stature, various skeletal abnormalities, severe dentinogenesis imperfecta, and insulin-dependent diabetes mellitus in four children.

These two genes are still not listed in the Nosology of genetic skeletal disorders despite these studies. Studies on a large cohort of patients using ES and WGS have, so far, notably, contributed to the molecular pathology of SD (7, 39–41) and have always contributed to the discovery of new genes responsible for SD, which lead to an increasing number of genes. This was presented by the increased number of identified genes in the updated revisions of the nosology of genetic skeletal disorders from 226 in 2010 (1) to 552 disease-causing genes that cover 737 skeletal disorders in the last revision in 2023 (6).

In addition to the genetic heterogeneity, there’s a large diversity of clinical manifestations of skeletal dysplasia. The recent update of the Nosology of genetic skeletal disorders reported 771 different disorders classified into 42 different groups (6). In our study, we categorized the detected disorders into four major groups: primary bone dysplasias, dysostosis, rare bone tumors, and lysosomal storage diseases. Primary bone dysplasias emerged as the most common and heterogeneous group in our cohort.

This clinical heterogeneity spanned diverse age categories within our cohort, with a particular emphasis on prenatal cases and carrier screening, both of which have significant implications for the healthcare system. This category showed a high diagnostic rate of 38.51% (n=181), with a positive yield of 23.2% (n=42) using both diagnostic approaches. Consistent with the findings of South America, fetal cases constitute 40% of all cases with genetic SDs (42). Using NGS, we achieved a diagnostic yield of prenatal diagnosis of 54.05% (n=20). Similar results using NGS for prenatal diagnosis were observed in the literature, summarized in the literature review, with an average positive diagnostic yield of 69% (43).

Fetal congenital anomalies not only increase infant morbidity and mortality but also cause intangible suffering for families. Ultrasound detection rates of fetal abnormalities are observed in 2–3% of pregnancies (44). Timely, accurate diagnoses and appropriate interventions for congenital anomalies are therefore crucial. Given that SDs are among the most common fetal malformations (45), prenatal diagnosis is essential. From a public health perspective, experts and policymakers have opted that newborn and carrier screening should be conducted as a state-run program, especially for rare diseases (46, 47). This will allow early detection of genetic conditions and congenital disorders, reducing health disparities and mortality at a young age and early parental decision-making (48).

Clinical and genetic diagnosis has an important impact on the patient’s clinical care and participates in initiating the appropriate patient treatment management. Recently, precise therapies such as gene therapies and authorized orphan drugs have been developed, particularly for rare diseases that are targeted to some specific altered genes. A definitive molecular diagnosis is essential for these specialized treatments, underscoring the role of genetic diagnostics in advancing precision medicine. To support potential therapeutic strategies for our patients, we examined the availability of specific medical interventions and orphan drugs associated with the identified diseases and genes. This analysis included resources such as the NHGRI Clinical Genomics Database (CGD) clinical categorization (49), Orphadata for orphan drugs (50), and RxGenes (51). We found registered drugs with orphan designation for 11 genes associated with different disorders, of which six are already in use as they have marketing authorization, with 2 of them using enzyme replacement therapy. In addition, gene therapy has been developed for five disorders. We also identified mechanism-based therapeutics, therapies targeting pathological mechanisms identified in specific diseases for five genes associated with different disorders. Finally, 31 disorders were identified with potentially beneficial interventions targeting certain clinical features, which could be advantageous for our patients.

In detail, we cite here examples of treatments for diseases that are highly presented in our cohort. The vosoritide is an approved drug for disorders related to *FGFR3* pathogenic variants (43, 52). Moreover, for this treatment, phase 2 and 3 clinical trials are currently ongoing for children between 0 to < 60 months and 5 to 18 years (2, 44). In our study, 38 patients present pathogenic variants in this gene, which could benefit from this treatment. The Asfotase alfa, which has been recently approved by the European Medicines Agency (EMA), should be started before the age of 5 years old for the most severe forms of hypophosphatasia (perinatal/infancy) (53). This could be an enzyme replacement therapeutic target for four patients in our cohort positively diagnosed. We cite also, the clinical trial started to evaluate the Denosumab treatment with moderate to severe OI use in children between the age of 5–10 years (ClinicalTrials.gov identifier: NCT01799798). It is also used as a potent and effective treatment for bone resorption pathologies such as osteoporosis and different types of bone tumors (54). OI is the most common disease detected in our patients (20 patients); in addition, infantile osteoporosis and osteochondromas are detected in 10 patients in our cohort. Hence, the discovery of the genetic etiology of our patients could help for better clinical management and treatment guidance for the referral clinicians and the patients.

The strength of our approach lies in the systematic implementation of next-generation sequencing within the national healthcare system, facilitating broad access to genetic testing. However, the generalizability of our findings regarding diagnostic yield and molecular pathology may be influenced by the unique genetic characteristics of the Slovenian population.In conclusion, this study outlines a national strategy for the genetic diagnosis of SDs in Slovenia, presenting data on the genetic epidemiology of these disorders within the Slovenian population and revealing substantial clinical and genetic variability. Our findings contribute to expanding the mutational spectrum by identifying novel variants and genes associated with extremely rare SD entities, supporting enhanced genotype–phenotype correlations. Furthermore, our experience underscores the utility of NGS for achieving molecular diagnoses and improving diagnostic yield. By integrating both SNV and CNV analyses from NGS data, our approach demonstrates a robust diagnostic yield, advocating for the inclusion of CNV analysis to aid in diagnosing unresolved cases. These genetic investigations refine clinical diagnostics and enable the application of emerging, targeted treatments including enzyme replacement therapy and gene therapy that show promising results for specific conditions, based on patient genetic profiles. Additionally, many clinical trials show promising treatments for skeletal dysplasia; however, further research is needed to refine and adapt these therapies, integrating genetic findings with therapeutic developments for broader patient benefit.

## Data Availability

The datasets presented in this study can be found in online repositories. The names of the repository/repositories and accession number(s) can be found in the article/**Supplementary Material**.
