# Supplementary material for: Unraveling the complexity of skeletal dysplasias in the national health system
Source: Front Endocrinol (Lausanne). 2025 Mar 10;16:1523737. doi: 10.3389/fendo.2025.1523737 (PMC11930811; doi:10.3389/fendo.2025.1523737)
Supplement: Supplementary file 1 [file Table1.docx]

Supplementary Material

Table 1 : Variants classified pathogenic or likely pathogenic associated to SD using NGS and direct gene sequencing

| *Gene* | Variant | Status | Type of variant | Inheritance | Disease |
| --- | --- | --- | --- | --- | --- |
| *ALPL* | c.542C>T, p.(Ser181Leu) / c.1166C>T, p.(Thr389Ile) | Compound Heterozygous | Missense variants | AR | Hypophosphatasia |
| *ALPL* | c.568_570del, p.(Asn190del) / c.1400T>C, p.(Met467Thr) | Compound Heterozygous | IN-frame Del / missense variant | AR | Infantile hypophosphatasia |
| *ALX4* | c.793C>T, p.(Arg265*) | Heterozygous | Nonsense | AD | Frontonasal dysplasia type 2 |
| *AMER1* | c.685dup, p.(Ala229Glyfs*2) | Heterozygous | Null frameshift | XLD | Osteopathy of the striatum with fetal craniosclerosis |
| *ANKH* | c.1124_1126del, p.(Ser375del) | Heterozygous | frameshift variant | AD | Craniometaphyseal dysplasia |
| *BRAF* | c.755G>C, p.(Arg252Pro) | Heterozygous | DE NOVO Missense variant | AD | Noonan syndrome 7 |
| *BRAF* | c.1502A>G, p.(Glu501Gly) | Heterozygous | missense variant | AD | Noonan syndrome 7 |
| *CBFB* | c.295_296dup, p.(Pro100Leufs*3) | Heterozygous | Null frameshift | AD | Cleidocranial dysplasia |
| *COL1A1* | c.727G>T, p.(Glu243*) | Heterozygous | Nonsense | AD | Osteogenesis imperfecta type 1 |
| *COL1A1* | c.1084G>A, p.(Gly362Ser) | Heterozygous | Missense variant | AD | Osteogenesis imperfecta |
| *COL1A1* | c.1678G>A, p.(Gly560Ser) | Heterozygous | Missense variant | AD | Osteogenesis imperfecta |
| *COL1A1* | c.1777G>A, p.(Gly593Ser) | Heterozygous | Missense variant | AD | Osteogenesis imperfecta IV |
| *COL1A1* | c.3168del, p.(Val1057Leufs*51) | Heterozygous | Null frameshift | AD | Osteogenesis imperfecta |
| *COL1A1* | c.2461G>A, p.(Gly821Ser) | Heterozygous | Missense variant | AD | Osteogenesis imperfecta |
| *COL1A1* | c.2490del, p.(Asp831Metfs*277) | Heterozygous | frameshift variant | AD | Osteogenesis imperfecta |
| *COL1A1* | c.3G>A, p.(Met1Ile) | Heterozygous | Missense variant | AD | Osteogenesis imperfecta type 1 |
| *COL1A2* | c.982G>A p.(Gly328Ser) | Heterozygous | Missense variant | AD | Osteogenesis imperfecta |
| *COL1A2* | c.551G>A, p.(Gly184Asp) | Heterozygous | Missense variant | AD | Osteogenesis imperfecta |
| *COL1A2* | c.1847G>A, p.(Gly616Glu) | Heterozygous | Missense variant | AD | osteogenesis imperfecta |
| *COL2A1* | c.3139G>A p.(Gly1047Ser) | Heterozygous | Missense variant | AD | Achondrogenesis, type II |
| *COL1A2* | c.577G>A, p.(Gly193Ser) | Heterozygous | Missense variant | AD | Osteogenesis imperfecta type 2 |
| *COL1A2* | c.1523G>A, p.(Gly508Asp) | Heterozygous | Missense variant | AD | Osteogenesis imperfecta type 2 |
| *COL1A2* | c.2306G>T, p.(Gly769Val) | Heterozygous | Missense variant | AD | Osteogenesis imperfecta |
| *COL1A2* | c.2629G>A, p.(Gly877Ser) | Heterozygous | Missense variant | AD | Osteogenesis imperfecta |
| *COL1A2* | c.1009G>A, p.(Gly337Ser) | Heterozygous | Missense variant | AD | Osteogenesis imperfecta |
| *COL1A2* | c.2549G>A, p.(Gly850Glu) | Heterozygous | missense variant | AD | Osteogenesis imperfecta |
| *COL1A2* | c.1972-2A>T | Heterozygous | splice site | AD | osteogenesis imperfecta II |
| *COL2A1* | c.1619G>T, p.(Gly540Val) | Heterozygous de novo | Missense variant | AD | Spondyloepiphyseal dysplasia |
| *COL2A1* | c.2356G>A p.(Gly786Ser) | Heterozygous | Missense variant | AD | Spondyloepiphyseal dysplasia congenita |
| *COL2A1* | c.2068G>A, p.(Gly690Arg) | Heterozygous | Missense variant | AD | Spondyloepiphyseal dysplasia congenita |
| *COL2A1* | c.1681G>A, p.(Gly561Ser) | Heterozygous | Missense variant | AD | Spondyloepiphyseal dysplasia congenita |
| *COL2A1* | c.1693C>T, p.(Arg565Cys) | Heterozygous | Missense variant | AD | Stickler syndrome |
| *COL2A1* | c.1286G>A, p.(Gly429Asp) | Heterozygous | Missense variant | AD | Congenital spondyloepiphyseal dysplasia |
| *COL2A1* | c.1957C>T, p.(Arg653*) | Heterozygous | nonsense | AD | Stickler syndrome |
| *COL10A1* | c.1923del, p.(Ile642Serfs*35) | Heterozygous | null frameshift | AD | Metaphyseal chondrodysplasia, Schmid type |
| *COL11A1* | c.4084_4101del, p.(Arg1362_Ala1367del) | Heterozygous | IN-frame DEL | AD | Stickler syndrome |
| *COL11A1* | c.2796_2813del, p.(Gln933_Pro938 del) | Heterozygous | In-frame del | AD | Stickler syndrome |
| *COL11A2* | c.4430G>T, p.(Gly1477Val) | Heterozygous | Missense variant | AD | Otospondylomegaepiphyseal dysplasia |
| *COMP* | c.983G>A, p.(Cys328Tyr) | Heterozygous | Missense variant | AD | Pseudoachondroplasia and epiphyseal dysplasia, multiple, 1 |
| *CREBBP* | c.6185_6195del, p.(Ile2062SerfsTer275) | Heterozygous | Frameshift variant | AD | Rubinstein-Taybi syndrome |
| *CREBBP* | c.5611A>C , p.(Thr1871Pro) | Heterozygous | Missense variant | AD | Rubinstein-Taybi syndrome |
| *CUL7* | c.3293T>G , p.(Leu1098Arg) | Homozygous | Missense variant | AR | 3-M syndrome 1 |
| *DYNC2H1* | c.7293-2_7298del, p.(Asp2431GlufsTer19) / c.7594C>T, p.(Arg2532Trp) | Compound Heterozygous | Null frameshift / Missense variant | AR | Short-rib thoracic dysplasia 3 with or without polydactyly |
| *DYM* | chr18:46,690,043-46,690,177 | Hemizygous | CNV | AR | Dyggve-Melchior-Clausen disease |
| *DVL1* | c.1607_1608dup, p.(Gly537Argfs*138) | Heterozygous | Null frameshift | AD | Robinow syndrome |
| *DVL1* | c.2006_2013del p.(Val669Glyfs*35) | Heterozygous | IN-frame DEL | AD | Robinow syndrome AD |
| *EFNB1* | chrX:68057909-68060002del (deletion of exons 2 to 4 ) | Heterozygous | CNV | AD | Craniofrontonasal dysplasia |
| *EP300* | c.7244A>G, p.(Ter2415Trpext*30) | Heterozygous | nonsense | AD | Rubinstein-Taybi syndrome |
| *ESCO2* | c.294_297del, p.(Arg99Serfs*2) | Homozygous | Null frameshift | AR | SC phocomelia syndrome Roberts syndrome |
| *EVC* | c.2894+3A>G, / c.1694del, p.(Ala565Valfs*23) | Compound Heterozygous | splice site / null frameshift | AR | Ellis-van Creveld syndrome |
| *EXT1* | c.1551G>A, p.(Trp517*) | Heterozygous | Nonsense | AD | Hereditary multiple osteochondromas |
| *EXT1* | c.1747C>T p.(Gln583*) | Heterozygous | Nonsense | AD | Hereditary multiple osteochondromas |
| *EXT1* | c.587_593del, p.(Tyr196Serfs*54) | Heterozygous | Null frameshift | AD | Hereditary multiple osteochondromas |
| *EXT1* | c.716dup, p.(Asp240Glyfs*49) | Heterozygous | Null frameshift | AD | Multiple osteochondromas |
| *EXT1* | c.1019G>A, p.(Arg340His) | Heterozygous | Missense variant | AD | Multiple osteochondromas |
| *EXT1* | c.2077del, p.(Ala693Leufs*13) | Heterozygous | Null frameshift | AD | Multiple osteochondromas |
| *EXT2* | c.639G>A, p.(Trp213*) | Heterozygous | nonsense | AD | Multiple osteochondromas |
| *EXT2* | c.866C>A, p.(Ser289*) | Heterozygous | nonsense | AD | Multiple osteochondromas |
| *FBN1* | c.3856del, p.(Leu1286*) | Heterozygous | Null frameshift | AD | Marfan syndrome |
| *FBN1* | c.5183C>T, p.(Ala1728Val) | Heterozygous | Missense variant | AD | Acromicric dysplasia |
| *FBN2* | c.3592T>C, p.(Cys1198Arg) | Heterozygous | Missense variant | AD | Beals syndrome |
| *FGFR2* | c.773C>G, p.(Ser258Cys) | Heterozygous | Missense variant | AD | Crouzon syndrome |
| *FGFR2* | c.862A>C, p.(Ile288Leu) | Heterozygous | Missense variant | AD | Crouzon syndrome |
| *FGFR2* | c.755C>G, p.(Ser252Trp) | Heterozygous | Missense variant | AD | Apert syndrome |
| *FGFR2* | c.758C>G, p.(Pro253Arg) | Heterozygous | Missense variant | AD | Apert syndrome |
| *FGFR2* | c.870G>T, p.(Trp290Cys) | Heterozygous | Missense variant | AD | Pfeiffer syndrome |
| *FGFR3* | c.742C>T, p.(Arg248Cys) | Heterozygous | Missense variant | AD | Thanatophoric dysplasia type 2 |
| *FGFR3* | c.746C>T, p.(Ser249Phe) | Heterozygous | Missense variant | AD | Thanatophoric dysplasia type 1 |
| *FGFR3* | c.746C>G, p.(Ser249Cys) | Heterozygous | Missense variant | AD | Thanatophoric dysplasia type 1 |
| *FGFR3* | c.1138G>C, p.(Gly380Arg) | Heterozygous | Missense variant | AD | Achondroplasia / hypochondroplasia |
| *FGFR3* | c.598C>T, p.(Arg200Cys) | Heterozygous | Missense variant | AD | Hypochondroplasia |
| *FGFR3* | c.749C>G, p.(Pro250Arg) | Heterozygous | Missense variant | AD | Muenke syndrome |
| *FGFR3* | c.1138G>A, p.(Gly380Arg) | Heterozygous | Missense variant | AD | Achondroplasia |
| *FGFR3* | c.1626C>A (p.Asn542Lys) | Heterozygous | Missense variant | AD | Hypochondroplasia |
| *FGFR3* | c.1620C>A (p.Asn540Lys) | Heterozygous | Missense variant | AD | Hypochondroplasia |
| *FGFR3* | c.1948A>G, p.Asn540Lys) | Heterozygous | Missense variant | AD | Hypochondroplasia |
| *FGFR3* | c.2420G>T, p.(Ter807Leuext*101) | Heterozygous | stop-loss variant | AD | Thanatophoric dysplasia type 2 |
| *FLNB* | c.644T>A, p.(Gly215Asp) | Heterozygous | Missense variant | AD | Atelosteogenesis type I |
| *FN1* | c.260G>A, p.(Cys87Tyr) | Heterozygous | Missense | AD | Spondylometaphyseal dysplasia, corner fracture type |
| *GJA1* | c.396_398del, p.(Ile132_Lys133delinsMet) | Heterozygous | IN-frame DEL | AD | Oculodentodigital dysplasia |
| *GJA1* | c.440T>C, p.(Met147Thr) | Heterozygous | missense variant | AD | Oculodentodigital dysplasia |
| *GJA1* | c.486G>C, p.(Lys162Asn) | Heterozygous | missense | AD | Oculodentodigital dysplasia |
| *GALNS* | c.860C>T, p.(Ser287Leu) | Homozygous | Missense variant | AR | Mucopolysaccharidosis IVA (Morquio syndrome A) |
| *GNAS* | c.127_129dup, p.(Pro43dup) | Heterozygous | Inframe dup | AD | Pseudohypoparathyroidism type 1A |
| *GNPTAB* | c.1514G>A, p.(Cys505Tyr) | Homozygous | Missense variant | AR | Mucolipidosis type III alpha/beta |
| *HOXD13* | c.820C>T, p.(Arg274*) / c.272C>G, p.(Ala91Gly) | Compound Heterozygous | Nonsense / missense | AD | Brachydactyly TYPE E |
| *HPGD* | c.263del, p.(Leu88Trpfs*7), /c.175_176del, p.(Leu59Valfs*8) | Compound Heterozygous | frameshift variants | AR | Hypertrophic osteoarthropathy, primary, autosomal recessive 1 |
| *HSPG2* | c.11913dup, p.(Lys3972Glufs*23) / c.11890G>A, p.(Gly3964Arg) | Compound Heterozygous | Null frameshift /Missense variant | AR | Schwartz-Jampel Syndrome |
| *HSPG2* | c.6440del, p.(Val2147Glyfs*25) / c.11566-25C>A | Compound Heterozygous | Null frameshift /Missense variant | AR | Schwartz-Jampel Syndrome |
| *KIF22* | c.443C>T, p.(Pro148Leu) | Heterozygous | Missense variant | AD | Spondyloepimetaphyseal dysplasia with joint laxity, type 2 |
| *LEMD3* | c.1700del, p.(Leu567*) | Heterozygous | Null frameshift | AD | Buschke-Ollendorff syndrome |
| *LEMD3* | c.2032C>T (p.Arg678*) | Heterozygous | Nonsense | AD | Buschke-Ollendorff syndrome |
| *LEMD3* | c.325C>T, p.(Arg109*) | Heterozygous | Nonsense | AD | Buschke-Ollendorff syndrome Osteopoikilosis |
| *LEMD3* | c.2011del, p.(Val671*) | Heterozygous | Null frameshift | AD | Osteopoikilosis |
| *LONP1* | c.2014C>T, p.(Arg672Cys) | Homozygous | Missense variant | AR | Cerebral, ocular, dental, auricular, and skeletal anomalies (CODAS) syndrome |
| *LRP5* | c.1850T>G, p.(Phe617Cys) | Heterozygous | Missense variant | AD | Osteopetrosis |
| *LZTR1* | c.401-2_401-1del / c.1889G>A, p.(Arg630Gln) | Compound heterozygous | Splice site/ missense variant | AR | Noonan syndrome |
| *MASP1* | c.992_993del, p.(Thr331Argfs*8) / c.1492dup, p.(Val498Glyfs*7) | Compound Heterozygous | frameshift variants | AR | 3MC syndrome 1 |
| *MIA3* | c.2768T>G, p.(Leu923*) | Homozygous | Nonsense | AR | Odotochondrodysplasia 2 with hearing loss and diabetes disease |
| *NF1* | c.5768C>A,p.(Thr1923Lys) | Heterozygous | Missense variant | AD | Noonan syndrome-Neurofibromatosis |
| *NF1* | c.2446C>, p.(Arg816*) | Heterozygous | Nonsense | AD | neurofibromatosis type 1 |
| *NFIX* | c.1473del,p.(Asn492Thrfs*105) | Heterozygous | Null frameshift | AD | Marshall-Smith syndrome |
| *NIPBL* | c.7459del, p.(Glu2487Lysfs*21) | Heterozygous | Null frameshift | AD | Cornelia de Lange syndrome |
| *NIPBL* | c.3534_3535del,p.(Lys1179Glufs6) | Heterozygous | Null frameshift | AD | Cornelia de Lange syndrome, type 1 |
| *NIPBL* | c.161T>A, p.(Leu54*) | Heterozygous | nonsense | AD | Cornelia de Lange syndrome |
| *PAPSS2* | c.121C>T, p.(Arg41*) | Homozygous | nonsense | AR | Brachyolmia 4 with mild epiphyseal and metaphyseal changes |
| *PHEX* | c.397C>T, p.(Gln133*) | Heterozygous | nonsense | XLD | X-linked hypophosphatemic rickets |
| *PLS3* | c.363C>G (p.Tyr121*) | Heterozygous | nonsense | XLD | X-linked osteoporosis with fractures |
| PLS3, SLC6A14 | Xq23(114799463_115670182)x0 | Homozygous | CNV | XLD | X-linked osteoporosis with fractures |
| *PLOD2* | c.1361G>T, p.(Gly454Val) / c.2038C>T, p.(Arg680) | Compound Heterozygous | Missense variant / Nonsense variant | AR | Bruck syndrome 2 |
| *PLOD2* | c.351dup, p.(Phe118fs*31) | Homozygous | Null frameshift | AR | Bruck syndrome 2 |
| *PTPN11* | c.417G>C, p.(Glu139Asp) | Heterozygous | Missense variant | AD | Noonan syndrome |
| *PTPN11* | c.922A>G, p.(Asn308Asp) | heterozygous | Missense variant | AD | Noonan syndrome |
| *PTPN11* | c.923A>G, p.(Asn308Ser) | Heterozygous | Missense variant | AD | Noonan syndrome |
| *RBM8A* | microdeletion of the 1q21.1 region encompassing the RBM8A gene | Hemizygous | CNV | AR | Thrombocytopenia-absent radius syndrome |
| *SALL1* | c.1027dup, p.(Ile343Asnfs*12) | Heterozygous | frameshift variant | AD | Townes-Brocks syndrome 1 |
| *SGSH* | c.197C>G, p.(Ser66Trp) / c.220C>T, p.(Arg74Cys) | Compound Heterozygous | Missense variants | AR | Mucopolysaccharidosis type IIIA (Sanfilippo syndrome A) |
| *SLCO2A1* | c.290G>A, p.(Arg97His) | Homozygous | Missense variant | AR | Hypertrophic osteoarthropathy, primary, autosomal recessive 2 |
| *SMARCAL1* | c.1687C>T, p.(Arg563*) / c.2542G>T, p.(Glu848*) | Compound Heterozygous | nonsense variants | AR | Schimke immuno-osseous dysplasia |
| *SMC1A* | c.802_804del, p.(Lys268del) | Heterozygous | IN-frame DEL | XLD | Cornelia de Lange syndrome 2 |
| *SMC1A* | c.1150A>G, p.(Lys384Glu) | Heterozygous | Missense variant | XLD | Cornelia de Lange syndrome 2 |
| *SOS1* | c.1300G>A, p.(Gly434Arg) | Heterozygous | Missense variant | AD | Noonan syndrome |
| *TBX6* | 16p11.2(29474877_30212207)x1 / c.1227G>A / g.30102802T>C / g.30103160 G>T | compound heterozygous | CNV, Haplotype of three SNP | AR | Spondylo-costal dysostosis type 5 |
| *TCF12* | c.1838G>A, p.(Arg613His) | Heterozygous | Missense variant | AD | Craniosynostosis type 3 |
| *TCF12* | 15p21.3(57523378-57579118)  (deletion of exons 9 to 19) | Hemizygous | CNV | AD | Craniosynostosis type 3 |
| *TCF12* | c.1541C>A, p.(Ser514*) | Heterozygous | nonsense | AD | Craniosynostosis, type 3 |
| *TPO* | (chr2:1476647_1497704del) and includes part of *TPO* gene | Heterozygous | CNV | AR | Thyroid dyshormonogenesis 2A |
| *TCIRG1* | c.2282del, p.(Gly761Alafs*22) / c.117+1G>A | Compound Heterozygous with c.117+1G>A | Null frameshift / splice site | AR | Infantile osteopetrosis |
| *TMEM38B* | chr9:108.444.592-108.478.48 del | Homozygous | CNV | AR | Osteogenesis imperfecta, type XVI |
| *TRPV4* | c.2396C>T, p.(Pro799Leu) | Heterozygous | Missense variant | AD | spondylometaphyseal dysplasia |
| *TRPV4* | c.1771T>A, p.(Tyr591Asn) | Heterozygous | Missense variant | AD | Spondylometaphyseal dysplasia |
| *TRPS1* | c.1230G>A, p.(Trp410* ) | Heterozygous | Nonsens | AD | Trichorhinophalangeal syndrome type 1 |
