# Supplementary material for: Unraveling the complexity of skeletal dysplasias in the national health system
Source: Front Endocrinol (Lausanne). 2025 Mar 10;16:1523737. doi: 10.3389/fendo.2025.1523737 (PMC11930811; doi:10.3389/fendo.2025.1523737)
Supplement: Supplementary file 2 [file Table2.docx]

Supplementary Material

Table 2: Novel variant detected in our patients with SD disorders

| **Patient** | ***Gene*** | **Variants** | **Type of variants** | **Status** | **Inheritance** | **Pathogenicity** | **Disease** |
| --- | --- | --- | --- | --- | --- | --- | --- |
| Patient 1 | *COL2A1* | c.2356G>A, p.(Gly786Ser) | Missense variant | Heterozygous | AD | Pathogenic | Spondyloepiphyseal dysplasia congenita |
| Patient 2 | *MASP1* | c.992_993del, p.(Thr331Argfs*8) RCV001199074 /  c.1492dup, p. (Val498Glyfs*7) RCV001199075 | Frameshift variants | Compound heterozygous | AR | Likely pathogenic | 3MC syndrome 1 |
| Patient 3 | *EFNB1* | chrX:68057909-68060002del | CNV | Heterozygous | AD | pathogenic | Craniofrontonasal dysplasia |
| Patient 4 | *DYNC2H1* | c.7293_7298del, p.(Asp2431fs) | Null frameshift | Compound heterozygous with c.7594C>T, p.(Arg2532Trp) | AR | Pathogenic / likely pathogenic | Short-rib thoracic dysplasia 3 with or without polydactyly |
| Patient 5 | *SALL1* | c.1027dup, p.(Ile343Asnfs*12) | frameshift variant | Heterozygous | AD | Pathogenic | Townes-Brocks syndrome 1 |
| Patient 6 | *COMP* | c.983G>A, p.(Cys328Tyr) | Missense variant | Heterozygous | AD | Likely pathogenic | Pseudoachondroplasia and epiphyseal dysplasia, multiple, 1 |
| Patient 7 | *PLOD2* | c.351dup, p.(Phe118Ilefs*31)  SCV001367200 | Null frameshift | Homozygous | AR | Likely pathogenic | Bruck syndrome 2 |
| Patient 8 | *HPGD* | c.263del, p.(Leu88Trpfs*7) | frameshift variant | Compound heterozygous with c.175_176del | AR | Likely pathogenic and pathogenic | Primary hypertrophic osteoarthropathy |
| Patient 9 | *COL1A2* | c.2629G>A, p.(Gly877Ser)  SCV001366261 | Missense variant | Heterozygous | AD | Likely pathogenic | Osteogenesis imperfecta |
| Patient 10 | *DVL1* | c.1607_1608dup, p.(Gly537Argfs*138) | Null frameshift | Heterozygous | AD | Likely pathogenic | Robinow syndrome |
| Patient 11 | *TCF12* | 15q21.3(57523378-57579118)x1 | CNV confirmed by MLPA | Heterozygous | AD | Likely pathogenic | Craniosynostosis type 3 |
| Patient 12 | *NFIX* | c.1473del, p.(Asn492ThrfsTer105) | Null frameshift | Heterozygous | AD | likely pathogenic | Marshall-Smith syndrome |
| Patient 13 | *COL10A1* | c.1923del, p.(Ile642Serfs*35) | Null frameshift | Heterozygous | AD | Likely pathogenic | Metaphyseal chondrodysplasia, Schmid type |
| Patient 14 | *AMER1* | c.685dup, p.(Ala229GlyfsTer2) | Null frameshift | Heterozygous | XLD | likely pathogenic | Osteopathy of the striatum with fetal craniosclerosis |
| Patient 15 | *COL11A2* | c.4430G>T, p.(Gly1477Val)  SCV000492909 | Missense variant | Heterozygous | AD | Likely pathogenic | Stickler syndrome |
| Patient 16 | *COL1A1* | c.3G>A, p.(Met1Ile) | Missense variant | Heterozygous | AD | Pathogenic | Osteogenesis imperfecta type 1 |
| Patient 17 | *PLOD2* | c.1361G>T, p.(Gly454Val)  SCV000492693 | Missense variant / Nonsense variant | Compound heterozygous withc.2038C>T, p.(Arg680Ter) | AR | likely pathogenic / pathogenic | Bruck syndrome 2 |
| Patient 18 | *COL2A1* | c.1286G>A,  p.(Gly429Asp) | Missense variant | Heterozygous | AD | Likely pathogenic | Spondyloepiphyseal dysplasia |
| Patient 19 | *GJA1* | c.486G>C, p.(Lys162Asn)  SCV001369329 | Missense variant | Heterozygous | AD | Likely pathogenic | Oculodentodigital dysplasia |
| Patient 20 | *HSPG2* | c.11913dup, p.(Lys3972GlufsTer23) / c.11890G>A, p.(Gly3964Arg) | Null frameshift /Missense variant | Compound heterozygous | AR | Pathogenic/likely pathogenic | Schwartz-Jampel Syndrome |
| Patient 21 | *NF1* | c.5768C>A, p.(Thr1923Lys) | Missense variant | Heterozygous | AD | Likely pathogenic | Neurofibromatosis type 1 |
| Patient 22 | *TCIRG1* | c.2282del, p.(Gly761AlafsTer22) | Null frameshift | Compound heterozygous with c.117+1G>A | AR | Likely pathogenic | Infantile osteopetrosis |
| Patient 23 | *CBFB* | c.295_296dup, p.(Pro100Leufs*3)  SCV002586990 | Null frameshift | Heterozygous | AD | Pathogenic | Cleidocranial dysplasia |
| Patient 24 | *CUL7* | c.920_929del p.(Leu307fs) | Null frameshift | Compound heterozygous with c.4886T>C, p.(Leu1629Pro) | AR | Pathogenic | 3-M syndrome 1 |
| Patient 25 | *NIPBL* | c.7459del, p.(Glu2487Lysfs*21)  SCV000747694 | Null frameshift | Heterozygous | AD | Pathogenic | Cornelia de Lange syndrome |
| Patient 26 | *NIPBL* | c.161T>A, p.(Leu54*)  SCV001366416 | Nonsense | Heterozygous | AD | Pathogenic | Cornelia de Lange syndrome |
| Patient 27 | *NIPBL* | c.3534_3535del, p.(Gln1178Glufs*6)  RCV001199109 | Null frameshift | Heterozygous | AD | Pathogenic | Cornelia de Lange syndrome, type 1 |
