# Supplementary material for: Unraveling the complexity of skeletal dysplasias in the national health system
Source: Front Endocrinol (Lausanne). 2025 Mar 10;16:1523737. doi: 10.3389/fendo.2025.1523737 (PMC11930811; doi:10.3389/fendo.2025.1523737)
Supplement: Supplementary file 3 [file Table3.docx]

Supplementary Material

Table 3: Novel variants classified as Hot or middle VUS detected in our patients with SD disorders

| **Patient** | ***Gene*** | **Variants** | **Type of variant** | **Status** | **Inheritance** | **Diseases** |
| --- | --- | --- | --- | --- | --- | --- |
| Patient 28 | *MIA3* | c.2768T>G, p.(Leu923*) | Nonsense | Homozygous | AR | Severe skeletal dyplasia phenotype |
| Patient 29 | *GDF5* | c.506C>T, p.(Pro169Leu) | Missense variant | Heterozygous | AD | Multiple Synostosis Syndrome Type 2 |
| Patient 30 | *CUL7* | c.4886T>C, p.(Leu1629Pro) | Missense variant | Compound heterozygous with c.920_929del p.(Leu307fs) | AR | 3-M syndrome 1 |
| Patient 31 | *CLCN7* | c.1688C>A, p.(Thr563Lys)  SCV001368602 | Missense variant | Heterozygous | AD | osteopetrosis type 2 |
| Patient 32 | *RMRP* | n.253T>G | Non-coding variant | Homozygous | AR | Cartilage-hair hypoplasia syndrome |
| Patient 33 | *CYP27B1* | c.1126G>A, p.(Glu376Lys) / c.974C>T, p.(Thr325Met) | Missense variants | Compound heterozygous | AR | Vitamin D-dependent rickets, type 1A |
| Patient 34 | *LONP1* | c.1939G>A, p.(Glu647Lys)  SCV001369580 | Missense variants | Compound heterozygous with c.2014C>T, p.(Arg672Cys) | AR | Cerebral, ocular, dental, auricular, and skeletal anomalies (CODAS) syndrome |
| Patient 35 | *NIPBL* | c.358G>A, p.(Gly120Arg) | Missense variant | Heterozygous | AD | Cornelia de Lange syndrome 1 |
| Patient 36 | *WDR19* | c.3874T>C (p.Cys1292Arg)  SCV001368493 | Missense variant | Compound heterozygous  c.291-12_291-11del | AR | Short rib–polydactyly syndrome |
